# Automated detection of fluorescent cells in in‐resin fluorescence sections for integrated light and electron microscopy

**DOI:** 10.1111/jmi.12700

**Published:** 2018-04-26

**Authors:** J. DELPIANO, L. PIZARRO, C.J. PEDDIE, M.L. JONES, L.D. GRIFFIN, L.M. COLLINSON

**Affiliations:** ^1^ School of Engineering and Applied Sciences Universidad de los Andes Santiago Chile; ^2^ Department of Computer Science University College London London United Kingdom; ^3^ Electron Microscopy The Francis Crick Institute London United Kingdom

**Keywords:** Cell segmentation, correlative light and electron microscopy, image processing, integrated light and electron microscopy, in‐resin fluorescence, watershed algorithm

## Abstract

Integrated array tomography combines fluorescence and electron imaging of ultrathin sections in one microscope, and enables accurate high‐resolution correlation of fluorescent proteins to cell organelles and membranes. Large numbers of serial sections can be imaged sequentially to produce aligned volumes from both imaging modalities, thus producing enormous amounts of data that must be handled and processed using novel techniques. Here, we present a scheme for automated detection of fluorescent cells within thin resin sections, which could then be used to drive automated electron image acquisition from target regions via ‘smart tracking’. The aim of this work is to aid in optimization of the data acquisition process through automation, freeing the operator to work on other tasks and speeding up the process, while reducing data rates by only acquiring images from regions of interest. This new method is shown to be robust against noise and able to deal with regions of low fluorescence.

## Lay description

Integrated Light and Electron Microscopy is a recent technique, which uses a light microscope inside an electron microscope to analyse the position of molecules in cells and tissues. The resulting data can give clues about the functions of those molecules in health and disease. However, this technique can produce huge amounts of image data, which has to be stored and analysed by scientists, with high computational and time costs. To streamline the process, we have designed a method whereby computers can automatically detect the position of the molecules of interest, using fluorescent labels, so that electron images are only acquired from these target regions. This method will form part of a workflow to automate the process of data collection, freeing scientists to work on other tasks.

## Introduction

Recent technological advances in electron microscopy have allowed the acquisition of extended volume data sets at high resolution (Peddie & Collinson, 2014). One of these methods is known as array tomography, whereby an array of ultrathin sections cut through resin‐embedded cells or tissues are imaged sequentially with a scanning electron microscope (SEM) to build up a 3D stack of images through the volume (Micheva & Smith, 2007; Wacker & Schroeder, 2013; Hayworth *et al*., 2014). In parallel, the field of correlative light and electron microscopy has enabled the mapping of functional information onto high‐resolution ultrastructural electron microscopy data, by detecting fluorescent biomarkers in the context of cell structure (Kopek *et al*., 2012; Bell *et al*., 2013; Löschberger *et al*., 2014; Johnson *et al*., 2015; Bykov *et al*., 2016; Mateos *et al*., 2016; Wolff *et al*., 2016). Integrated light and electron microscopy (ILEM) (Liv *et al*., 2013) combines both microscopes in one device by placing a light microscope inside the vacuum chamber of an electron microscope. It is possible to perform integrated array tomography inside the ILEM using in‐resin fluorescence (IRF) sections, in which both fluorescent and electron signals have been preserved (Peddie *et al*., 2014, 2017). This technique delivers data from both modalities with almost perfect alignment.

Both modalities – light and electron microscopy – produce enormous amounts of data that must be handled and processed using novel techniques. ILEM has the potential to reduce the volume of data acquired, by using the fluorescent signal to target regions of interest (ROI) for subsequent electron microscopy imaging, a process designated ‘smart tracking’. This kind of approach would save valuable time for researchers by automating the process of integrated array tomography.

Software‐assisted array tomography can significantly help a microscopist in the procedures aimed at 3D digitization of a sample. A software package based on a multiscale approach has been developed (Hayworth *et al*., 2014) for array tomography using SEM, directing the mapping and imaging of selected regions across a library of sections. This software package, WaferMapper, can manage the process of converting sections from an automatic tape‐collecting ultramicrotome tape, into an image volume. An initial low‐resolution mapping step is suggested, using either an optical image or an electron microscopy montage of the entire wafer. A second step requires more detailed low‐resolution images and is done automatically in the SEM. For use in ILEM, this and further steps should be revised, because the electron beam destroys the fluorescence signal and therefore cannot be used for mapping purposes.

A recent review covered the free software tools for detection of fluorescence cells in light micrographs (Wiesmann *et al*., 2015). The authors studied and tested 12 image analysis tools, including Icy (De Chaumont *et al*., 2012), CellProfiler (Carpenter *et al*., 2006), ImageJ/Fiji (Schneider *et al*., 2012; Schindelin *et al*., 2012) and Omero (Allan *et al*., 2012). A test user with a life sciences background was asked to segment four fluorescence micrographs with all the tools. For two challenging data sets, they report that the best results were achieved using a seeded watershed approach. However, these tests were not performed using ultrathin IRF sections.

Techniques for IRF yield fluorescence images with high variability in intensity in each cell. This is partially due to inherent variability in fluorescence expression levels, and partially as a result of ultrathin sectioning which reduces the number of fluorescent molecules available for detection per cell per section. Furthermore, the wide variability in cell shape due to the sectioning process makes the detection problem even harder.

Here, we present a novel algorithm workflow for smart tracking of fluorescent cells in IRF sections in the ILEM for semiautomated ILEM.

### Contributions

- This paper presents a method for detection and localization of fluorescent cells in ultrathin IRF sections, and makes it available as a Matlab program with a graphical user interface.^1^ To the best of our knowledge, there are no other specialized methods that target this specific problem.
- There is no standard ground truth for this problem. Our results show that the manual segmentations by expert microscopists are highly subjective. Therefore, we show the results of measuring algorithm‐expert and inter‐expert variability.
- Our method is simple and uses standard tools that are available in most software packages and can be easily implemented.
- The method introduced in this paper is shown to be robust against noise and can deal with regions of low fluorescence intensity.

## Results

### Image acquisition in the ILEM

**Figure 1:**
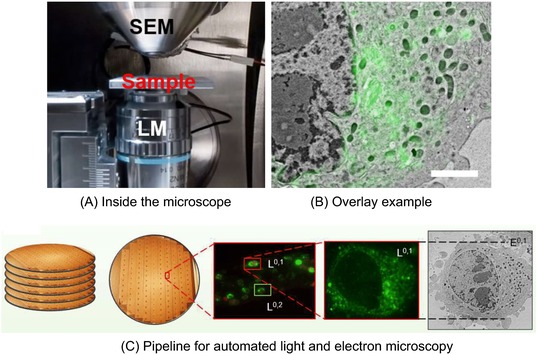
Workflow for correlative and integrated light and electron microscopy. (A) Close up of vacuum chamber inside an integrated light and electron microscope. Modified from Liv *et al*. (2013). (B) An example of an overlay of the fluorescence and EM data. Scale bar: 2μm. (C) Suggested pipeline for automated light and electron microscopy.

**Figure 2:**
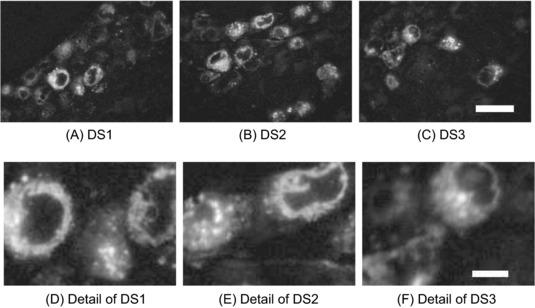
Widefield fluorescence micrographs of fluorescent cells in ultrathin IRF sections. (A–C) Fluorescence micrographs for an ultrathin section in the data sets DS1, DS2, DS3, corresponding to three specimens. The scale bar in (C) is 20μm long. (D–F) Zoomed‐in cells from (A–C). Scale bar in (f): 5μm.

In ILEM, light and electron images are obtained in the same microscope, with the light and electron beams aligned to the same axis (Haring *et al*., 2017) (Fig. 1A). The resulting images need almost no postacquisition alignment (Fig. 1B). Therefore, the fluorescence image can be used to locate cells for electron imaging in an automated correlative pipeline (Fig. 1C). Serial ultrathin sections from IRF blocks containing HeLa cells expressing GFP‐C1 were imaged using the widefield fluorescence microscope inside the electron microscope chamber (Peddie *et al*., 2014), giving three data sets for subsequent algorithm development (DS1, DS2 and DS3) (Fig. 2).

### Expert segmentation as ground truth

**Figure 3:**
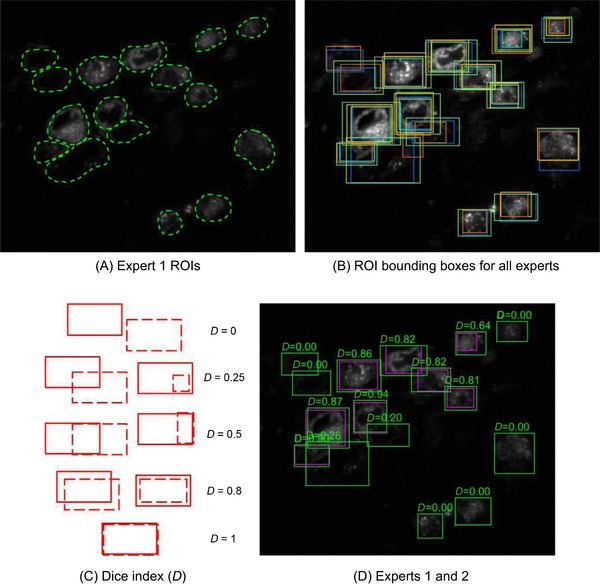
Ground truth segmentation of cell images. (A) Free‐hand ROI segmentation of HeLa cells by Expert 1. (B) Bounding boxes for the cell segmentations by the five experts were superimposed, giving one colour to each of the experts, showing the variability and subjective nature of their work. (C) The Dice index (*D*, see definition in the main text) between two rectangular ROIs is a measure of how good a detection is, considering both a correct localization and size. (D) The Dice index allows for a quantification of how subjective two expert segmentations are. Here, if Expert 1 is considered as the ground truth, the average Dice between segmentations by Expert 1 and Expert 2 is ${\bar{D}}_{\mathrm{GT}}=45\%$.

To provide ground truth data, five expert microscopists were asked to segment the entirety of all GFP‐positive cells in two data sets (DS1 and DS2) by drawing around the edge of the fluorescent cytoplasmic signal in each image. The level of expertise of each microscopist varied across a wide spectrum; some were familiar with IRF images, whereas others were not. The expert microscopists found an average of 14.1 cells per slice image. The segmentation took 1.4 h per expert on average, with the group delivering more than 100 cell segmentations per hour (Fig. 3A). It was immediately obvious that there was large variation in segmentations between experts (Fig. 3B), and so comparisons were made of performance within the expert group before using the segmentations as ground truth to judge the performance of automated cell detection algorithms. The Dice index was used for numeric evaluation of the difference between expert segmentations (Fig. 3C). The goal of this exercise was not to assess the segmentations of each expert, but to study the prior knowledge involved and the subjective component of their work. The mean interexpert DICE was only 67.7% on average (Fig. 3D and Table 1), demonstrating that the problem is difficult to tackle even for experts.

**Table 1:** Average Dice when comparing segmentations by experts (DS2, image 1). Results under 60% are shown in bold text

	Expert 1	Expert 2	Expert 3	Expert 4	Expert 5
Expert 1	100%	**45%**	74%	**55%**	80%
Expert 2	83%	100%	71%	70%	85%
Expert 3	83%	**45%**	100%	**54%**	70%
Expert 4	85%	**56%**	71%	100%	86%
Expert 5	79%	**44%**	63%	**55%**	100%

### Semi‐automated segmentation using Ilastik

**Figure 4:**
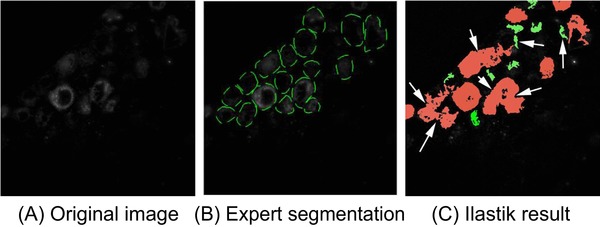
Semiautomated segmentation of cells using Ilastik. All the pixels from the manual segmentation of one expert were used as training examples for Ilastik random forests. (A) Original image for DS1 ‐ slice 3. (B) Segmentation by Expert 1. (C) Ilastik result. White arrows show some examples of two types of errors: some groups of two or three close cells were merged as one and some cell parts were marked as ‘not a cell’ (green).

Ilastik (Sommer *et al*., 2011) was used to segment the fluorescent cells, to test the performance of current state‐of‐the‐art software for semi‐automated image segmentation (Fig. 4). For this experiment, the manual segmentation by one expert (see example in Fig. 4B) was chosen as training data for pixel classification. Each segmented pixel was considered an example of the class ‘cell’, and all the other pixels in the slice were considered examples of the class ‘not a cell’. Although Ilastik was able to segment the cells in the images, there were two main classes of errors, where adjacent cells were merged and where some cell regions were marked as ‘not a cell’ (Fig. 4C). Though these errors may be corrected by further refinement, the interaction required in seeding the segmentation and in correcting errors rules out the use of this semi‐automated detection method for automated on‐the‐fly detection during imaging as part of a smart‐tracking correlative pipeline. We therefore moved to develop an algorithm that would automatically detect fluorescent cells in IRF images without additional user interaction.

### Design and performance of new automated segmentation algorithm

**Figure 5:**
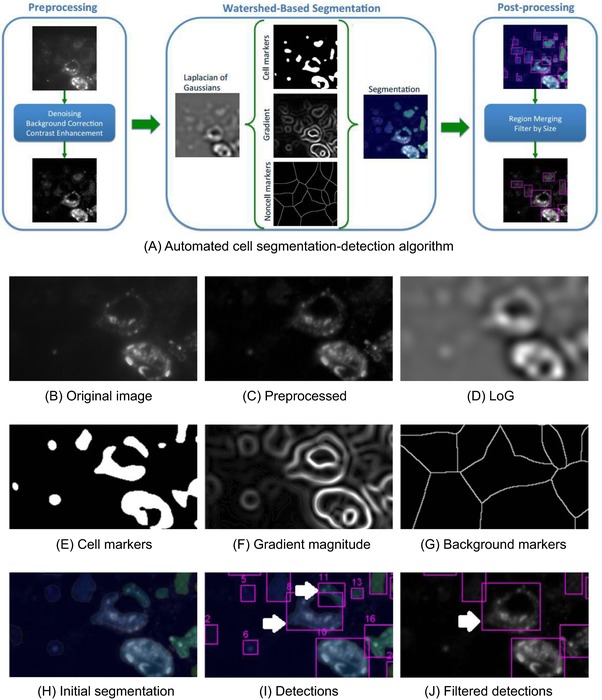
Development of automated cell segmentation workflow, based on the watershed algorithm, and partial results of algorithm steps. (A) Steps of the algorithm. (B) Raw image. (C) Preprocessed image. (D) Laplacian of Gaussian (LoG) image feature. (E) Cell pixel markers. (F) Gradient of the image feature. (G) Background pixel markers. (H) The watershed transform of modified gradient, superimposed transparently on the image. (I) Bounding boxes for watershed labels. Arrows show two boxes corresponding to over segmentation in one cell. (J) Bounding boxes for watershed labels, after merging and size filtering. The arrow shows a box coming from merging of two labels.

A workflow was designed for automated segmentation of fluorescent cells in IRF images (Fig. 5), consisting of pre‐processing steps to remove noise and artefacts, a watershed‐based algorithm to detect and segment the cells, and post‐processing steps to clean up the resulting segmentations (Fig. 5A). The raw image (Fig. 5B) was pre‐processed to remove noise (an optional step), and then uneven illumination was removed by background correction and enhancement of contrast (Fig. 5C). A linear filter was used as a feature to highlight the borders of the ring‐like objects that result from the selected sample and staining (Fig. 5D). Markers for the presence of cells were obtained as the brightest pixels in the feature image and saved (Fig. 5E). Two more results were fed as inputs for the main watershed step: the gradient magnitude of the image feature (Fig. 5F) and candidate ‘non cell’ pixels (Fig. 5G). The initial segmentation that results (Fig. 5H) is converted into its bounding boxes (Fig. 5I) and detections that are too large or small are filtered, whereas merging detections that correspond to the same cell (Fig. 5J).

**Figure 6:**
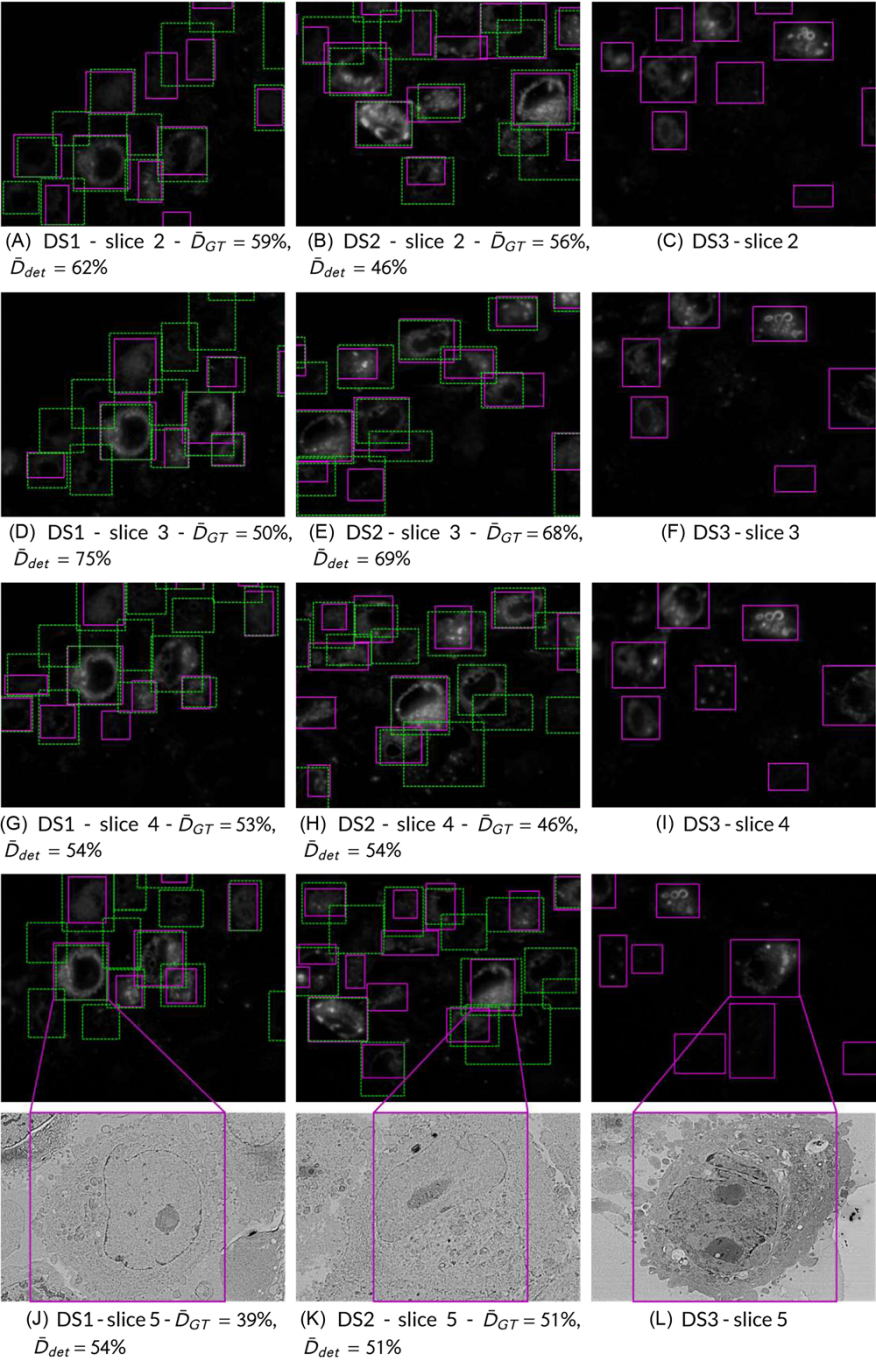
Results of detection for part of the HeLa cell data sets. Detections in solid magenta rectangles and ground truth in dashed green boxes. Left (A, D, G, J): Some slices of data set DS1. Centre (B, E, H, K): Slices of DS2. Right (C, F, I, L): DS3, no ground truth available.

The algorithm was applied to detect cells in all three data sets (Fig. 6). The Dice index was used to compare the output of the automated segmentation algorithm (cell detections) to manual segmentations by the expert microscopists (ground truth). Figures 6(A, D, G and J) show the results for data set DS1, with Dice calculated against one of the expert microscopists. The Dice index ranges from 39% to 75%. Figures 6(B, E, H and K) show the same results for DS2 where the Dice index varies from 46% to 69%. Figures 6(C, F, I and L) show the output of our method for DS3, where no ground truth is available. Therefore, no Dice values can be obtained. Whereas the mean interexpert DICE was 67.7%, the mean algorithm‐expert DICE was 68.08% (DS2, image 1), indicating that the algorithm performs at least as well as expert microscopists (Table 2). The cell detections by our watershed‐based algorithm achieved an average Dice index of 58%. The global average recall of 67% means that our algorithm can find 67% of the objects in the ground truth, with D>50%. A global average precision of 63% is interpreted as 63% of our detections being correct and with D>50% (Table 3).

**Table 2:** Average Dice for sample segmentation results. Results for data set DS2, image 1, are shown. See comments in main text

	Expert 1	Expert 2	Expert 3	Expert 4	Expert 5	Avg.
Image 1	64%	73%	64%	77%	62%	68.08%

**Table 3:** Average performance for our segmentation results

	Global Avg.
Dice	58%
Recall	67%
Precision	63%

### Development of a synthetic data set to model other fluorophore distributions

**Figure 7:**
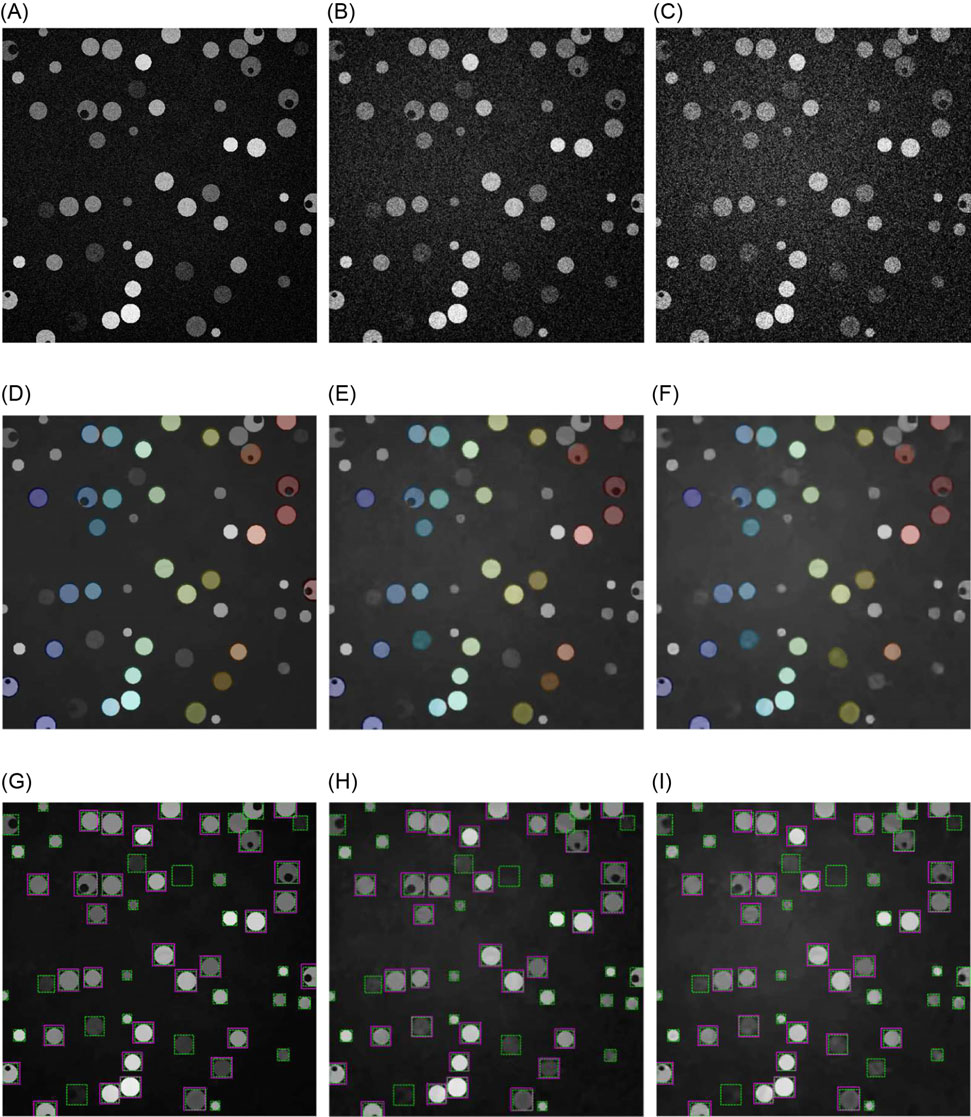
Watershed‐based detection steps and dependence on noise level for simulated data. (A–C) Simulated fluorescence images with Gaussian noise and σ=20, and magnitude of bell‐shaped background $A_{bg}=80$. A rolling ball filter was used to level the background. (B) Same configuration, but Gaussian noise with σ=40. (C) σ=60. (D–F) Watershed‐based segmentations, from the simulated images in (A–C). (G–I) Watershed‐based detections (solid magenta) and ground truth (dashed green).

As larger amounts of ILEM data become available, it will become feasible to start applying recent machine learning techniques, such as convolutional neural networks and deep learning. However, a large number of parameters implies a need for a large number of training examples. In the absence of real training data, a data set synthesized from a small amount of real data was developed, similar to the ‘flying chairs’ data set used for learning of optical flow (Dosovitskiy *et al*., 2015). The synthetic cell data set developed for this purpose was a simplistic representation of the cell population. A representative slice from a simulated fluorescence data set modelling a cytoplasmic expression pattern with a slice thickness of 100 nm is shown (Figs. 7A–C). Based on our prior knowledge of the ILEM images, slices were corrupted with Gaussian noise with standard deviations σ=20 (Fig. 7A), σ=40 (Fig. 7B) and σ=60 (Fig. 7C). We then applied our watershed‐based method to these images (Figs. 7D–F) and compared the ground truth (green) and automated segmentation results (magenta) for the three images (Figs. 7G–I). Recall (*r*) for the three noise levels was between 86% and 88%. The precision (*p*) range was 94–98%. This is a controlled experiment and the measures here are much better than for the real data, as expected for a simulated data set which is a simple representation of the real problem. However, the simulated data set will expedite further algorithm development against different fluorophore patterns (for example, nuclear or punctate fluorophore localizations), noise levels, cell shape and density.

## Discussion

Our work shows that it is possible to automatically locate ROI in ultrathin IRF sections, using the fluorescence signal to identify cells for subsequent electron imaging.

Though the current state‐of‐the‐art in shallow learning platforms, Ilastik, was able to identify fluorescent cells, it required ground truth training data from an expert microscopist as well as postcorrection to separate joined cells and to reclassify small dim regions of fluorescence as cells, making it unsuitable for on‐the‐fly detection of cells in an automated correlative workflow.

For detection and definition of a region of interest for electron imaging in ILEM, it is better to err on the side of caution. Acquiring more data than needed is preferred to missing fluorescent cells, since subsequent exposure to the electron beam destroys the fluorescence signal in all cell in the field of view. This leads to some criteria to weight detection errors. A large region of interest that contains an actual cell should be preferred over a region of interest that misses part of a cell. Likewise, a false positive detection where there is no cell should be preferred over missing the detection of a cell. Regarding recall (*r*) and precision (*p*), we should aim at having *r* as close to 1 as possible, as a top priority. As a second priority, *p* should be as close to 1 as possible. Even operating with a conservative ‘if in doubt, image it’ policy, this could lead to a significant reduction in the total area to be imaged, with concomitant savings in data storage and processing requirements.

The cell detections by our watershed‐based algorithm achieved an average Dice index of 58%, which was an improvement over the performance of some expert microscopists. Indeed, the interexpert Dice scores show that identification of fluorescent cells in ultrathin IRF sections is a difficult problem, even for humans. Though it is natural to expect some differences in ground truth segmentation results between experts, our results showed that agreement was surprisingly poor. The Dice scores revealed two groups of experts, which in postanalysis proved to be those with biology and physics backgrounds. The scientists with a biology background had a deeper understanding of the type of cells in the sample and were able to find cells that look very dim in the data, or that were smaller than expected due to being a glancing section through the edge of the cell.

For individual images in the data sets, interexpert Dice scores reached an average of 67.7%, whereas mean algorithm‐expert Dice score reached 68.08%, indicating that the algorithm can perform as well as experts on some images. However, algorithm performance could undoubtedly be improved. As we gather IRF images from a greater variety of cell types and fluorescent labels, it will be possible to incorporate additional fluorescence patterns into the algorithm to increase robustness for multiple biological applications. In addition, 3D information gathered from sequential serial sections will allow us to identify the central slice for each cell, which would usually be the largest area for a cell with a roughly spherical shape, and use this position to track the cell outwards through the adjacent sections to capture and increase confidence in smaller, dimmer cell edges. Further development and implementation of the IRF cell simulation model will expedite this process in the absence of real data.

Using the current algorithm, and future iterations that may include machine learning approaches, we foresee a workflow for automated integrated array tomography, whereby automated detection of fluorescent cells drives automated acquisition of electron images from cells of interest, which will speed up discovery research while minimizing the cost and compute resource required for 3D correlative microscopy by focusing data acquisition to specific ROI.

## Materials and methods

### Sample preparation

HeLa cells expressing a cytoplasmic GFP‐C1 fluorescent tag were embedded as described previously (Peddie *et al*., 2014). Sections of 200‐nm thickness were cut using an ultramicrotome and collected on indium‐tin oxide‐coated glass cover slips, which are conductive and optically transparent.

### Data acquisition

ILSEM was performed on the same day as sectioning using a SECOM light microscope platform (Delmic B.V., Delft) with Nikon Plan Apo 40x/0.95 objective, mounted on a Quanta 250 FEG SEM (FEI Company, Eindhoven). GFP fluorescence was stimulated by excitation with a 488‐nm laser light source and multiband filters (Di01‐R405/488/594 dichroic, FF01‐446/532/646‐25 emission; Semrock, Rochester, NY, USA). Individual fluorescence images at an XY pixel resolution of 178 nm were collected from matched areas of five serial sections using an EMCCD camera (iXon 897 Ultra; Andor Technology, Belfast, U.K.). The exposure time was 2 s, and power density was 0.5 W/cm^2^ at the sample level. For fluorescence imaging, the chamber was maintained at a partial pressure of 200 Pa, created using water vapour. To collect matching SEM images of specific cells of interest at an XY pixel resolution of 16.5 nm, the system was pumped to high vacuum ($∼10^{-3}$ Pa). The vCD backscatter detector (FEI Company, Eindhoven) was used at a working distance of 5.8 mm, and inverted contrast images were acquired (2.5 keV, spot size 3.5, 30 μm aperture and pixel dwell time of 60 μs for a 1536 * 1103 pixel image frame).

### Manual segmentation

Manual segmentation was performed using a touchscreen interface (Wacom) in Fiji (Schindelin *et al*., 2012; Schneider *et al*., 2012) with the freehand selection tool, and exported as ROIs from the ROI manager. Five microscopists, with varied expertise in light and electron microscopy, segmented five images each from two data sets (DS1 and DS2).

### Semiautomated segmentation using Ilastik

For pixel classification in Ilastik, six features were selected: Gaussian smoothing, Laplacian of Gaussian, Gaussian gradient magnitude, difference of Gaussians, structure tensor eigenvalues and Hessian of Gaussian eigenvalues. Random forests were trained for those features with ground truth data from one of the expert microscopists.

### Development of automated workflow

Our workflow for automated detection of cells was implemented in Matlab and is available for download https://github.com/jdelpiano/irfCellSegmentation. A graphical user interface was developed for easy access of the workflow to users with no experience in programming. The workflow consisted of the following steps. First, an optional denoising step may be applied to images if required. Due to its excellent results in tests with simulated fluorescence, the Matlab implementation of the block‐matching and 3D filtering algorithm was used (Dabov *et al*., 2007), assuming a good estimation of the noise level sigma. Widefield fluorescence microscopy images of IRF sections tend to have a Gaussian‐shaped background intensity, which was reliably corrected with the rolling ball filter. Due to the sample preparation procedures and fluorescent protein expression levels, some cells were very dim in the fluorescence image, and therefore contrast limited adaptive histogram equalization (CLAHE) (Zuiderveld, 1994) was applied to increase the intensity of the dimmer cells. Three scalar fields were fed to the watershed transform as preparation for segmentation: (1) A threshold of 92% was used in the cumulative density function to obtain the 8% brightest pixels in the feature image and save them as candidate cell pixels, (2) the gradient magnitude for the feature image, which was the result of applying a Laplacian of Gaussian linear filter with parameter σ=7 to the preprocessed image and (3) markers for background or absence of cells. The markers for background were obtained from the image complement of the cell markers. The distance transform was applied to that complement and then the watershed transform was used to find the skeleton of the structures observed in the distance transform.

### Dice index for inter‐expert and algorithm‐expert comparisons

To quantify comparison of cell segmentations, we defined the Dice index (*D*) between two segmentations as

(1) D=(2A∩B)/(A+B),

where *A* and *B* were the areas of the corresponding segmentations, and A∩B is the area of the intersection of both detections.

Given a set of cell detections and a set of ground truth segmentations, a computer does not know which detected cell corresponds to which cell in the ground truth. Therefore, the calculation of the Dice index between them results in a matrix which may recall a confusion matrix. We need to define the Dice index for each element in that matrix as

(2) $$D_{\mathrm{ij}}=\left(2A_{i}\cap B_{j}\right)/\left(A_{i}+B_{j}\right),$$

where $A_{i}$ and $B_{j}$ are the areas of the *i*th detection and the *j*th ground truth detection, and $A_{i}\cap B_{j}$ is the area of the intersection of both detections. A Dice index matrix can be defined by ${\left\{D_{\mathrm{ij}}\right\}}_{i=1,\ldots ,N_{\det};j=1,\ldots ,N_{\mathrm{GT}}}$, where $N_{\det}$ is the number of detections and $N_{\mathrm{GT}}$, the number of objects in the ground truth set.

There are two intuitive choices for an average Dice, given by Eqs (3) and (4), one with respect to the detections that have been found ${\bar{D}}_{\det}$ and one with respect to the ground truth ${\bar{D}}_{\mathrm{GT}}$. These choices are not equivalent nor symmetric.

(3) $$\begin{array}{ccc} {\bar{D}}_{\det} & = & \frac{1}{N_{\det}}\sum_{i=1}^{N_{\det}}\max_{j}\left\{D_{\mathrm{ij}}\right\}, \end{array}$$

(4) $$\begin{array}{ccc} {\bar{D}}_{\mathrm{GT}} & = & \frac{1}{N_{\mathrm{GT}}}\sum_{j=1}^{N_{\mathrm{GT}}}\max_{i}\left\{D_{\mathrm{ij}}\right\}. \end{array}$$

As we aim at defining the field of view for obtaining high‐resolution microscopy data, Dice indexes will be determined over the rectangular bounding boxes for each ground truth or estimated segmentation.

A cell segmentation will be considered correct as a detection if the Dice index *D* between itself and a ground truth cell detection is greater than a threshold, which was defined for the experiments shown here as 0.5. To analyse the performance of cell detection, we define two more quantities: recall *r* and precision *p*, as

(5) $$\begin{array}{c} r=\frac{\mathrm{TP}}{\mathrm{TP}+\mathrm{FN}}, \end{array}$$

(6) $$\begin{array}{c} p=\frac{\mathrm{TP}}{\mathrm{TP}+\mathrm{FP}}, \end{array}$$

where *TP* is the number of true positives, which are the correct detections of cells; *FN*, the number of false negatives, corresponds to the ground truth objects that were not detected and *FP* is the number of false positives. TP+FN is the number of objects in the ground truth data set.

### Development of a synthetic data set to model other fluorophore distributions

In order to test the detection of various fluorescent patterns, simulated cell data were generated with a custom MATLAB program that randomly placed cells within the volume. Each spherical cell had its size and brightness drawn from a Gaussian distribution, and a cytoplasmic staining was represented by a uniform intensity inside the sphere apart from an empty spherical region denoting the nucleus. On top of this, Gaussian noise was added at σ=20,40,60 (Figs. 7A–C). The resulting volume was sliced into dimensions typical for this type of experiment.

To obtain a ground truth, a bounding box was calculated for each simulated object, before adding Gaussian noise with standard deviation σ=20,40,60. Figures 7(A–C) show the result of preprocessing these images.

## Footnotes

1
https://github.com/jdelpiano/irfCellSegmentation.
